# Changes in headache characteristics with oral appliance treatment for obstructive sleep apnea

**DOI:** 10.1038/s41598-021-82041-6

**Published:** 2021-01-28

**Authors:** Ji Woon Park, Sujay Mehta, Sandra Fastlicht, Alan A. Lowe, Fernanda R. Almeida

**Affiliations:** 1 Department of Oral Health Sciences, Faculty of Dentistry, University of British Columbia, 2199 Wesbrook Mall, Vancouver, BC V6T 1Z3 Canada; 2 Department of Oral Medicine and Oral Diagnosis, School of Dentistry and Dental Research Institute, Seoul National University, 101, Daehak-ro, Jongno-gu, Seoul, 03080 Republic of Korea; 3 Vancouver Orofacial Pain, 1160 Burrard Street #701, Vancouver, BC V6Z 2E8 Canada

**Keywords:** Diseases, Health care, Medical research, Signs and symptoms

## Abstract

Changes in headache characteristics in obstructive sleep apnea (OSA) patients following oral appliance treatment was investigated for the first time. Thirteen OSA patients with headaches treated with a mandibular advancement device were investigated. Level I polysomnography and Migraine Disability Assessment Questionnaire were completed before and after treatment. Various headache characteristics and concomitant conditions were analyzed. The patient was considered a headache responder when ≥ 30% reduction in headache frequency following treatment. Differences in headache and polysomnographic parameters were compared between headache responder groups. Eight patients (62%) were headache responders. Eleven patients (85%) before and 7 (54%) after treatment reported morning headaches. Significantly more patients had bilateral headache in the responder group before treatment (P = 0.035). The severest headache intensity (P = 0.018) at baseline showed a significant decrease in the headache responder group after treatment. The time spent in N2 (r = − 0.663, P = 0.014), REM sleep (r = 0.704, P = 0.007) and mean oxygen saturation (r = 0.566, P = 0.044) showed a significant correlation with post-treatment average headache intensity. Pre-treatment lower PLM index (r = − 0.632, P = 0.027) and higher mean oxygen saturation levels (r = 0.592, P = 0.043) were significantly correlated with higher post-treatment severest headache intensity. Treatment with an oral appliance is beneficial for many OSA patients with headaches. It should be considered as an alternative treatment in headache patients with mild to moderate OSA.

## Introduction

Obstructive sleep apnea (OSA) is caused by repetitive obstruction of the upper airway that results in complete or partial cessation of airflow. OSA is known to afflict 3–20% of the general population and is showing a dramatic increase along with its socioeconomic burden over the last years^1–4^. OSA is associated with various clinical symptoms such as snoring, excessive daytime sleepiness, fatigue, neurocognitive impairment, cardiovascular disease, endocrinological problems, nocturia, nocturnal sweating, psychological problems, and also headache^5,6^. The importance of headache as a clinical finding of OSA has been recognized as sleep apnea headache was included in the International Classification of Headache Disorders under the heading of headache attributed to disorder of homeostasis and headache attributed to hypoxia and/or hypercapnia^7^. The prevalence of headaches in OSA patients has been reported to range from 15 to 60%^8–13^. However, there is still controversy whether patients with OSA experience more headaches compared to the general population and whether OSA severity is associated with the frequency and intensity of headache episodes. Several studies show higher headache prevalence in patients with OSA and an association with OSA severity^13–15^, while others were not in line with such findings^16,17^. The underlying mechanism by which OSA causes headaches has not been fully elucidated. Vibration from snoring, intermittent hypoxia, hypercapnia, arousals, sleep fragmentation, disturbances in cerebral blood flow regulation, and transient intracranial pressure increases have been suggested as possible etiologic factors^16,18^. The treatment of OSA has shown to alleviate sleep-related headaches. Such results could be reproduced in refractory headache patients with continuous positive airway pressure (CPAP) treatment even when the severity of OSA was mild^19,20^.

All previous studies evaluating the effect of OSA treatment on headache characteristics are concentrated on CPAP as the sole treatment modality. However, long-term CPAP adherence is unsatisfactory with over 30% of the initially adherent patients failing to use CPAP at 5 years from initiation^21^. Based on such observations dental approaches including oral appliances (OA) are recommended as promising alternatives in OSA management in the latest guidelines^22^. Growing evidence implies that even severe OSA can be efficiently treated with OA that protrude the mandible to maintain upper airway patency and decrease its collapsibility through various mechanisms^23^. In spite of the increasing use of OA for the treatment of OSA, its effects on headaches have not been previously evaluated.

Therefore, the aim of this study was to investigate the change in headache prevalence and characteristics in consecutive OSA patients confirmed with level 1 polysomnography (PSG) that were treated with OA. Furthermore, we aimed to determine which clinical and PSG related variables may predict the improvement in headaches in response to OA treatment for OSA.

## Results

A total of 13 headache patients (four males, nine females) who were referred for OA therapy for the treatment of OSA completed pre- and post-treatment headache evaluation questionnaires and full night PSGs. Demographic characteristics are shown in Table 1 with the entire sample and separating the patients depending on headache frequency after OA treatment. Among the six patients that showed a decrease in average headache intensity post-treatment, the decrease in VAS was 2.67 ± 1.86 (mean ± SD). Three patients (23% of the total patients) showed ≥ 50% decrease in average headache intensity and ≥ 50% decrease in MIDAS score with OA treatment.

**Table 1:** Baseline demographic and clinical characteristics of study groups.

Variables	All (n = 13)	Headache responder (n = 8)	Headache non-responder (n = 5)	P-value
Age (years)^a^	49.92 (9.53)	48.13 (10.44)	52.80 (8.07)	0.413
Gender (male/female)^b^	4/9 (44%)	3/5 (38%)	1/4 (20%)	0.506
BMI (kg/m^2^)^a^	27.79 (4.93)	25.99 (4.06)	31.40 (4.97)	0.070
Ethnicity (Caucasian/Asian/unknown)^b^	10/1/2	6/1/1	4/0/1	0.428
Alcohol drinker^b^	9/13 (69%)	6/8 (75%)	3/5 (60%)	0.310
Current smoker^b^	1/13 (8%)	0/8 (0%)	1/5 (20%)	0.217
Hypertension^b^	3/13 (23%)	1/8 (12%)	2/5 (40%)	0.252
Abnormal cardiac rhythm^b^	1/13 (8%)	0/8 (0%)	1/5 (20%)	0.217
OSA responder^b^^,c^	5/8 (63%)	3/5 (60%)	2/3 (67%)	0.714
OA treatment adherence^b^^,d^	9/13 (69%)	6/8 (75%)	3/5 (60%)	0.510

*BMI* body mass index, *OSA* obstructive sleep apnea, *OA* oral appliance.

^a^Differences between groups were tested with t-test: Mean (SD).

^b^Differences between groups were tested with Chi-square test: number of male or positive participants/total number of participants (percentage of male or positive participants).

^c^Decrease in AHI > 50% compared to baseline AHI. Results given as number of OSA responders/total number of patients (percentage of OSA responders).

^d^Using the appliance for > 50% of the night on ≥ 4 nights a week. Results given as number of OA adherent patients/total number of patients (percentage of OA adherent patients).

Possible confounders including age, gender, BMI, ethnicity, smoking, alcohol intake, and cardiovascular conditions did not show any significant differences between the groups. More patients in the headache responder group were adherent to their OA treatment compared to the non-responder group although the difference was not statistically significant.

### Headache characteristics of headache responder and non-responder groups

Eight patients (62% of the total patients) showed headache symptoms that were consistent with migraine and five patients (38% of the total patients) could be primarily diagnosed as tension type headache pre-treatment. The average intensity of a headache episode was 4.58 ± 1.64 (mean ± SD) on a 0–10 VAS scale and the average duration was 128.04 ± 80.88 (mean ± SD) mins per episode pre-treatment. Bright lights, loud noise, stress, sleep deprivation, TMJ, and neck pain were included in the reported aggravating factors. Eleven patients (85% of the total patients) reported to have morning headaches. Three patients (23% of the total patients) had unilateral headache, five patients (38% of the total patients) had concomitant nausea/vomiting, six patients (46% of the total patients) had concomitant autonomic symptoms, and ten patients (77% of the total patients) reported increased sensitivity (light/sound/smell) with headaches before OA treatment.

**Figure 1:**
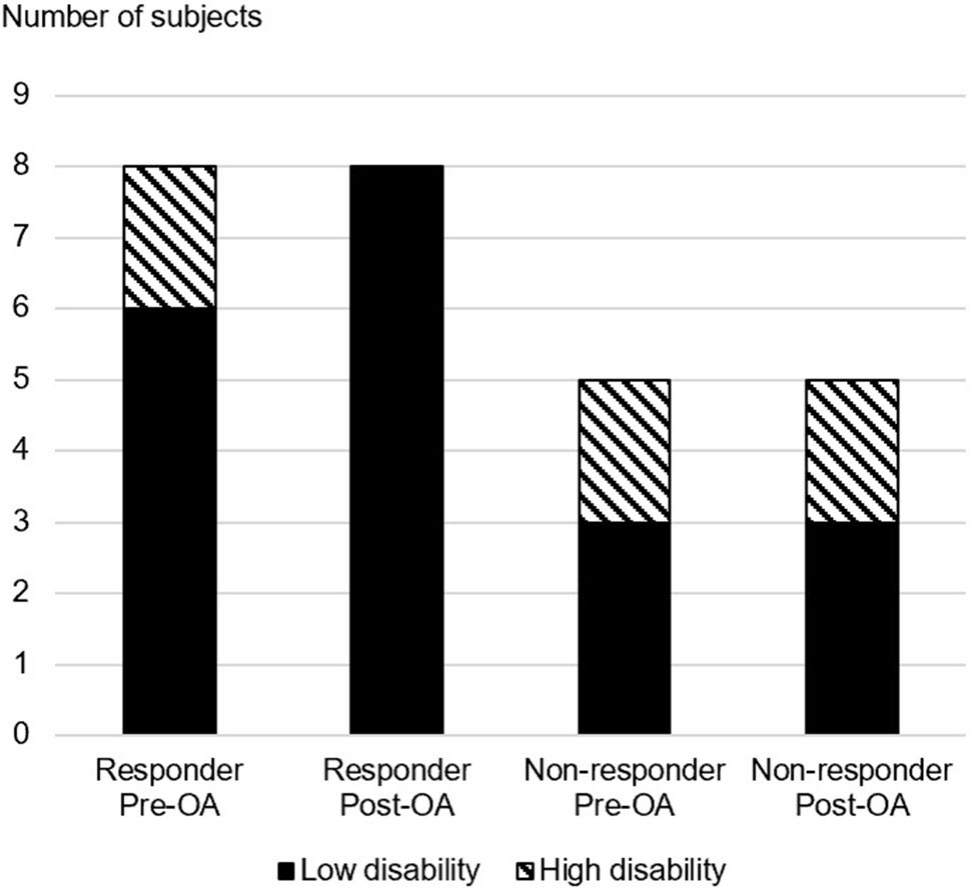
Change in distribution of MIDAS disability groups with oral appliance treatment. Low disability (MIDAS score 0–10), High disability (MIDAS score ≥ 11). *OA* oral appliance. Microsoft PowerPoint 2019 (Microsoft, Redmond, WA, USA) https://www.microsoft.com/en-ca/microsoft-365/powerpoint.

**Figure 2:**
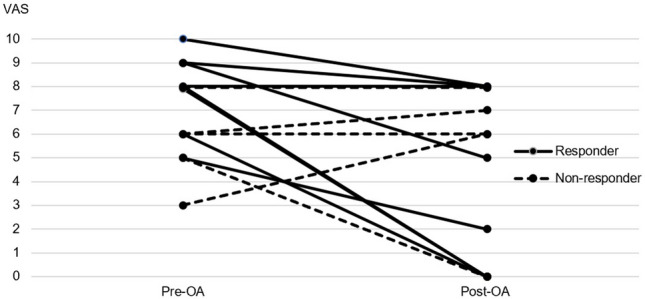
Change in severest headache intensity for each individual subject of the headache responder and non-responder groups. *VAS* visual analog scale (0 is no pain and 10 is the worst imaginable pain possible). *OA* oral appliance. Microsoft PowerPoint 2019 (Microsoft, Redmond, WA, USA) https://www.microsoft.com/en-ca/microsoft-365/powerpoint.

There were significantly more patients with bilateral headache in the headache responder group compared to the non-responder group (P = 0.035) pre-treatment. More patients in the headache responder group showed less disability due to headache after OA treatment as shown in Fig. 1. The severest intensity (P = 0.018) and frequency (P = 0.011) of headaches showed a significant decrease only in the headache responder group post-treatment. Two patients in the headache non-responder group showed an increase in severest headache intensity after treatment as illustrated in Fig. 2. The average intensity of headaches decreased 13% in the headache responder group but increased in the headache non-responder group. The presence of morning headaches showed a decrease in both groups with OA treatment. Concomitant nausea/vomiting and increased sensitivity also showed a tendency to decrease in both groups post-treatment.

Descriptive values of types and symptoms of headaches are summarized in Table 2.

**Table 2:** Headache characteristics according to headache responder group.

Variables	Headache responder (n = 8)		Headache non-responder (n = 5)	
	Pre-OA	Post-OA	Pre-OA	Post-OA
Frequency (days/3 month)^a^	6.00 (4.25–11.50)**	1.50 (0.00–2.75)**	1.00 (0.50–75.00)	3.00 (0.50–75.00)
Type (migraine, tension-type)^b^^,d^	5/8 (63%)	1/8 (13%)	3/5 (60%)	1/5 (20%)
Intensity (average)^c^^,e^	4.75 (1.75)	4.13 (3.14)	4.40 (1.52)	4.60 (2.88)
Intensity (current)^c^^,e^	2.13 (2.85)	0.38 (1.06)	3.20 (2.17)	2.40 (3.36)
Intensity (when severest)^c^^,e^	7.88 (1.64)**	4.43 (3.74)**	5.60 (1.82)	5.40 (3.13)
Duration of episode (mins)^c^	158.57 (110.52)	192.86 (254.47)	97.50 (51.23)	120.00 (81.24)
Morning headache^b^^,d^	6/8 (75%)	4/8 (50%)	5/5 (100%)	3/5 (60%)
MIDAS score^a^	1.00 (0.00–12.00)	1.00 (0.00–4.25)	0 (0.00–35.00)	0 (0.00–75.00)
MIDAS grade^b,^^d^	6/8 (75%)	8/8 (100%)	3/5 (60%)	3/5 (60%)
Localization (unilateral, bilateral)^b^^,d^	8/8 (100%)*	0/8 (0%)	2/5 (40%)*	2/5 (40%)
Nausea/Vomiting^b^^,d^	3/8 (38%)	1/8 (13%)	2/5 (40%)	1/5 (20%)
Sensitivity (light, sound, smell)^b^^,d^	6/8 (75%)	3/8 (38%)	4/5 (80%)	3/5 (60%)
Autonomic symptoms^b^^,d^	4/8 (50%)	3/8 (38%)	2/5 (40%)	3/5 (60%)

Grouping was based on ≥ 30% reduction in headache frequency.

*OA* oral appliance.

*Significant difference: P < 0.05, comparison between headache responder and non-responder.

**Significant difference: P < 0.05, comparison between pre- and post-oral appliance treatment.

^a^Differences between groups were tested with Mann–Whitney test and pre- and post-OA data were tested with Wilcoxon Rank-sum test: Median (lower quartile-upper quartile).

^b^Differences between groups were tested with Chi-square test and pre- and post-OA data were tested with McNemar’s test.

^c^Differences between groups were tested with t-test and pre- and post-OA data were tested with paired t-test: Mean (SD).

^d^Results given as number of patients with migraine, morning headaches, low disability, bilateral headache, nausea (vomiting), sensitivity, autonomic symptoms/total number of patients (percentage of patients with each characteristic).

^e^Based on a visual analog scale, 0 (no pain) − 10 (worst pain imaginable).

### Polysomnographic characteristics of headache responder and non-responder groups

The average AHI was 15.40 events/h (10.95–31.10) before and 7.2 events/h (3.15–10.20) after treatment. Two patients showed resolution of OSA, four showed treatment success, one showed suboptimal success, and one resulted in treatment failure. Five patients (63%) were OSA responders with a decrease in AHI > 50% compared to before treatment.

Table 3 illustrates the PSG characteristics of headache responders and non-responders. The AHI (P = 0.030), hypopnea index (HI) (P = 0.002), supine AHI (P = 0.030), NREM AHI (P = 0.045) were significantly lower in the headache responder group before treatment. Significantly more patients had mild OSA in the responder group (P = 0.017). The decrease in AHI was significant only in the headache responder group following OA treatment (P = 0.043). HAR increased in both groups following OA treatment. The mean and longest duration of apneic and hypopneic events only decreased in the headache responder group following treatment. There was a significant decrease in lowest oxygen saturation only in the headache non-responder group following treatment (P < 0.001).

**Table 3:** Polysomnographic characteristics according to headache responder group-respiratory parameters.

Variables	Headache responder		Headache non-responder	
	Pre-OA (n = 8)	Post-OA (n = 5)	Pre-OA (n = 5)	Post-OA (n = 3)
AHI (events/h)^a^	11.45 (10.20–15.23)*^,^**	6.30 (1.85–7.65)**	24.20 (20.30–42.05)*	11.00 (6.90–11.00)
AI (events/h)^a^	3.60 (1.13–6.68)	0.40 (0.05–1.43)	6.20 (1.00–11.60)	3.15 (1.50–3.15)
HI (events/h)^a^	8.05 (7.75–8.78)*	3.85 (1.60–7.00)	22.20 (16.60–31.05)*	17.00 (5.50–17.00)
OSA severity^b^^,d^ (mild/moderate/severe)	7/0/1*	5/0/0	0/3/2*	2/0/1
Supine AHI (events/h)^a^	12.00 (10.00–15.60)*	3.70 (1.48–8.40)	43.50 (22.90–54.41)*	15.55 (7.70–15.55)
Non-supine AHI (events/h)^a^	11.10 (8.50–23.65)	2.90 (1.15–23.15)	21.65 (14.83–32.07)	5.76 (5.00–5.76)
Positional OSA^b^^,d^	0/8 (0%)	2/5 (40%)	1/5 (20%)	1/3 (33%)
REM AHI (events/h)^a^	16.05 (4.85–34.00)	3.90 (0.58–11.95)	35.40 (6.20–67.65)	30.65 (9.10–30.65)
NREM AHI (events/h)^a^	11.05 (5.75–15.60)*	3.35 (1.05–8.35)	20.40 (16.60–40.90)*	16.50 (6.60–16.50)
REM-related OSA^b^^,d^	3/8 (38%)	2/5 (40%)	3/5 (60%)	1/3 (33%)
HAR^a^	2.23 (1.29–4.32)	3.59 (2.67–3.59)	3.52 (1.81–9.61)	4.80 (3.67–4.80)
Mean apnea duration (s)^c^	22.29 (6.92)	12.88 (9.15)	15.46 (9.45)	21.00 (2.26)
Longest apnea duration (s)^c^	43.81 (27.58)	16.10 (11.84)	27.58 (21.71)	43.00 (11.03)
Mean hypopnea duration (s)^a^	22.05 (19.55–25.80)	18.95 (18.05–27.65)	20.30 (17.10–25.70)	25.50 (21.80–25.50)
Longest hypopnea duration (s)^a^	46.25 (40.85–51.85)	29.15 (25.25–69.65)	38.90 (33.40–80.05)	63.45 (61.50–63.45)
Mean oxygen saturation (%)^a^	96.64 (1.93)	95.62 (2.03)	96.38 (1.32)	94.73 (1.58)
Lowest oxygen saturation (%)^a^	87.88 (5.08)	89.70 (4.69)	86.72 (3.60)**	86.50 (0.71)**
Oxygen saturation < 90% (%TST)^b^	0.10 (0.00–0.67)	0.10 (0.00–0.50)	0.20 (0.00–1.24)	0.75 (0.70–0.75)

*OA* oral appliance, *AHI* apnea hypopnea index, *AI* apnea index, *HI* hypopnea index, *REM* rapid eye movement sleep, *NREM* non rapid eye movement sleep, *HAR* hypopnea/apnea ratio, *RAR* respiratory effort related arousal index + hypopnea/apnea ratio.

*Significant difference: P < 0.05, comparison between headache responder and non-responder.

**Significant difference: P < 0.05, comparison between pre- and post-oral appliance treatment.

^a^Differences between groups were tested with Mann–Whitney test and pre- and post-OA data were tested with Wilcoxon Rank-sum test: Median (lower quartile-upper quartile).

^b^Differences between groups were tested with Chi-square test and pre- and post-OA data were tested with McNemar’s test.

^c^Differences between groups were tested with t-test and pre- and post-OA data were tested with paired t-test: Mean (SD).

^d^Results given as number of patients with mild, moderate, severe, positional, REM-related OSA/total number of patients (percentage of patients with each characteristic).

The polysomnographic sleep architecture data are shown in Table 4. The respiratory arousal index was significantly lower in the headache responder group before treatment (P = 0.019). There was a significant decrease in the percentage of NREM 1 sleep only in the headache non-responder group following treatment (P < 0.001). There was a significant increase in PLM index in both headache responder (P = 0.020) and non-responder (P < 0.001) groups after treatment.

**Table 4:** Polysomnographic characteristics according to headache responder group-sleep architecture, oxygen saturation, limb movement, and sleepiness.

Variables	Headache responder		Headache non-responder	
	Pre-OA (n = 8)	Post-OA (n = 5)	Pre-OA (n = 5)	Post-OA (n = 3)
Total Sleep Time (mins)^a^	362.14 (53.70)	363.95 (56.63)	321.72 (136.96)	375.63 (27.52)
Sleep efficiency (%TST)^a^	82.88 (9.67)	82.75 (10.95)	82.30 (13.51)	85.80 (4.40)
Sleep latency (mins)^b^	17.95 (6.93–22.90)	7.95 (2.63–13.58)	13.90 (2.35–33.60)	15.50 (2.80–15.50)
REM latency (mins)^b^	79.50 (48.00–161.00)	84.75 (69.50–145.75)	93.75 (46.63–221.50)	87.50 (74.50–87.50)
WASO (mins)^b^	49.50 (17.58–78.93)	71.00 (20.35–114.83)	23.50 (11.15–64.15)	59.05 (35.00–59.05)
Spontaneous arousal index^b^	1.25 (0.68–3.20)	3.60 (1.70–5.35)	1.80 (0.05–25.50)	8.10 (1.00–8.10)
Respiratory arousal index^b^	9.95 (4.53–15.23)*	4.70 (1.65–7.68)	20.50 (19.90–132.15) *	20.10 (6.90–20.10)
N1 sleep (%TST)^a^	9.86 (7.03)	12.73 (10.33)	13.42 (8.01)**	9.5 (8.77)**
N2 sleep (%TST)^a^	69.25 (14.32)	62.48 (8.65)	65.52 (11.36)	65.23 (4.21)
Slow wave sleep (%TST)^b^	0.70 (0.00–4.43)	0.35 (0.00–3.25)	1.20 (0.05–8.25)	2.20 (1.80–2.20)
REM sleep (%TST)^b^	21.35 (10.50–26.23)	22.70 (18.85–29.25)	19.30 (4.20–29.75)	22.70 (14.50–22.70)
PLM index^a^	14.35 (14.90)**	27.55 (33.89)**	19.98 (12.32)**	30.35 (11.67)**
ESS^b^	6.00 (3.25–14.75)	–	13.00 (9.00–15.00)	–
ESS group^c^^,d^	3/8 (38%)	–	4/5 (80%)	–

*OA* oral appliance, *TST* total sleep time, *REM* rapid eye movement sleep, *WASO* wake after sleep onset, *N* non rapid eye movement sleep, *PLM* periodic limb movement, *ESS* Epworth Sleepiness Scale.

*Significant difference: P < 0.05, comparison between headache responder and non-responder.

**Significant difference: P < 0.05, comparison between pre- and post-oral appliance treatment.

^a^Differences between groups were tested with t-test and pre- and post-OA data were tested with paired t-test: Mean (SD).

^b^Differences between groups were tested with Mann–Whitney test and pre- and post-OA data were tested with Wilcoxon Rank-sum test: Median (lower quartile-upper quartile).

^c^Differences between groups were tested with Chi-square test.

^d^Results given as number of patients with excessive daytime sleepiness (ESS > 10)/total number of patients (percentage of patients with excessive daytime sleepiness).

### Headache characteristics of oral appliance adherent and non-adherent groups

**Figure 3:**
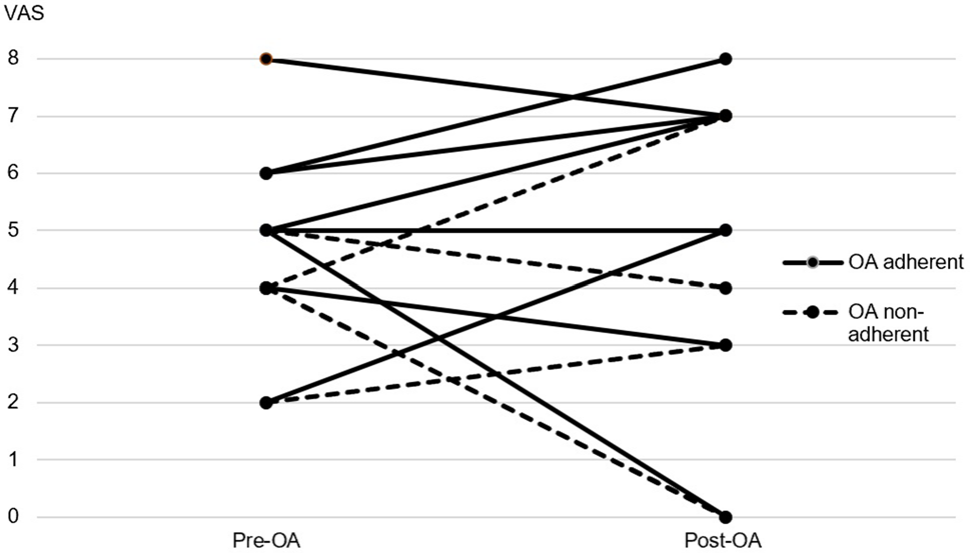
Change in average headache intensity for each individual subject of the oral appliance adherent and non-adherent groups. *OA* oral appliance. Microsoft PowerPoint 2019 (Microsoft, Redmond, WA, USA). https://www.microsoft.com/en-ca/microsoft-365/powerpoint.

As described in Table 5, the headache frequency showed a significant decrease following treatment only in the OA adherent group (P = 0.024). The severest headache intensity showed a notable decrease while the average headache intensity increased for five patients following OA treatment as seen in Fig. 3. Less patients had concomitant autonomic symptoms in the OA-adherent group before treatment (P = 0.021). More patients had migrainous headache in the non-adherent group after treatment. The intensity of headaches and the prevalence of morning headaches both decreased following OA treatment in both groups however more patients in the non-adherent group reported persistent morning headaches and more frequent headaches after treatment. MIDAS score tended to be higher and more patients had concomitant symptoms after OA treatment in the non-adherent group.

**Table 5:** Headache characteristics according to oral appliance adherence group.

Variables	OA adherent (n = 9)		OA non-adherent (n = 4)	
	Pre-OA	Post-OA	Pre-OA	Post-OA
Frequency (days/3 month)^a^	6.00 (2.50–11.00)**	1.00 (0.00–3.00)**	37.00 (1.75–85.00)	2.50 (0.50–68.25)
Type (migraine or tension-type)^b,d^	5/9 (63%)	1/9 (11%)	3/4 (75%)	1/4 (25%)
Intensity (average)^c^^,e^	5.00 (1.66)	4.67 (3.04)	3.75 (1.26)	3.50 (2.89)
Intensity (current)^c,e^	2.00 (2.87)	0.56 (1.67)	3.75 (1.26)	2.50 (3.32)
Intensity (when severest)^c,e^	7.33 (2.24)	5.38 (3.50)	6.25 (1.26)	3.75 (3.30)
Duration of episode (mins)^c^	138.75 (111.03)	222.86 (242.40)	130.00 (45.83)	67.50 (51.23)
Morning headache^b,d^	7/9 (78%)	4/9 (44%)	4/4 (100%)	3/4 (75%)
MIDAS score^a^	0.00 (0.00–9.00)	0.00 (0.00–5.50)	9.00 (0.00–27.00)	2.50 (0.00–68.75)
MIDAS grade^b,d^	7/9 (78%)	8/9 (89%)	2/4 (50%)	3/4 (75%)
Localization (unilateral or bilateral)^b,d^	1/9 (11%)	1/9 (11%)	2/4 (50%)	1/4 (75%)
Nausea/Vomiting^b,d^	3/9 (33%)	1/9 (11%)	2/4 (50%)	1/4 (75%)
Sensitivity (light/sound/smell)^b,d^	6/9 (67%)	4/9 (44%)	4/4 (100%)	2/4 (50%)
Autonomic symptoms^b,d^	2/9 (22%)*	3/9 (33%)	4/4 (100%)*	3/4 (75%)
Headache responder^b,d,f^	6/9 (67%)		2/4 (50%)	

*OA* oral appliance.

*Significant difference: P < 0.05, comparison between OA adherent and non-adherent.

**Significant difference: P < 0.05, comparison between pre- and post-oral appliance treatment.

^a^Differences between groups were tested with Mann–Whitney test and pre- and post-OA data were tested with Wilcoxon Rank-sum test: Median (lower quartile-upper quartile).

^b^Differences between groups were tested with Chi-square test and pre- and post-OA data were tested with McNemar’s test.

^c^Differences between groups were tested with t-test and pre- and post-OA data were tested with paired t-test: Mean (SD).

^d^Results given as number of patients with migraine, morning headaches, low disability, unilateral headache, nausea (vomiting), sensitivity, autonomic symptoms, headache responder/total number of patients (percentage of patients with each characteristic).

^e^Based on a visual analog scale, 0 (no pain) − 10 (worst pain imaginable).

^f^Those with ≥ 30% reduction in headache frequency were considered headache responder.

### Correlation between pre- and post-oral appliance treatment headache parameters and pre-treatment polysomnographic parameters

An increase in time spent below 90% oxygen pre-treatment showed a significant correlation with lower pre-treatment average headache intensity (r = − 0.571, P = 0.041). A decrease in the longest apnea duration (r = − 0.568, P = 0.043), total sleep time (r = − 0.698, P = 0.008), and sleep efficiency (r = − 0.693, P = 0.009), and a higher PLM index (r = 0.553, P = 0.050) showed a significant correlation with higher pre-treatment current headache intensity. The increase in spontaneous arousal index (r = 0.562, P = 0.046) and decrease in PLM index (r = − 0.627, P = 0.022) showed a significant correlation with higher pre-treatment severest headache intensity. An decrease in PLM index (r = − 0.604, P = 0.049) showed a significant correlation with longer pre-treatment headache episode duration. Less time spent in NREM 2 (r = − 0.663, P = 0.014) and more in REM sleep (r = 0.704, P = 0.007) and higher mean oxygen saturation (r = 0.566, P = 0.044) showed a significant correlation with higher post-treatment average headache intensity. Higher mean oxygen saturation (r = 0.592, P = 0.043) and lower PLM index (r = − 0.632, P = 0.027) showed a significant correlation with higher post-treatment severest headache intensity. Higher total sleep time (r = 0.654, P = 0.029) and sleep efficiency (r = 0.719, P = 0.013) showed a significant correlation with longer post-treatment headache episode duration. More time spent in NREM 1 (r = 0.562, P = 0.046) and less in NREM 2 sleep (r = − 0.692, P = 0.009) showed a significant correlation with higher post-treatment headache frequency.

## Discussion

This is the first study to evaluate the effect of OA treatment on headaches in OSA patients. There is good literature on OSA, headaches and the effects of treatment, but all previous studies were based on CPAP treatment. Notably, all OSA patients in this study were diagnosed based on level 1 sleep studies to verify the effect of sleep related parameters on headache characteristics. The results of the current study show that the majority of evaluated patients experienced a significant decrease in headache frequency following OA treatment. This underlies the fact that headache should be routinely considered in the diagnostic process of OSA and treatment based on OA should be actively applied to those for the betterment of not only OSA but also headache severity.

A previous study reported that among the 33 OSA patients using CPAP, 39% reported a greater than 50% improvement in headache severity and frequency^20^. However, this result is incomparable to our study since the aforementioned study consisted of a higher percentage of severe OSA patients and the criteria for headache improvement were different. The results of our study showed that 62% of patients experienced a greater than 30% reduction in headache frequency. The main analysis in our study was based on such criteria for equal distribution of each headache responder groups. When using a greater than 50% reduction in MIDAS score or 50% reduction in average headache severity as the criteria for treatment responder, 23% of the patients could be differentiated as headache responders independently. Such a value is lower compared to the results based on CPAP. This discrepancy may result from the difference in efficacy of each treatment modality in controlling OSA symptoms that are directly related to the severity of headache. OA treatment is generally less efficacious than CPAP in improving polysomnographic parameters of OSA, especially in regard to AHI and oxygen saturation^24^. However, the underlying mechanism through which OSA and headaches are linked are not fully revealed so it is difficult to directly quantify the effect of OSA alleviation on headache improvement^16,18^. Furthermore, recent studies show that to achieve favorable long-term health outcomes, adherence should also be considered in evaluating the final effectiveness of a treatment. Studies show that OA treatment may not be inferior to CPAP in improving cardiovascular outcomes and health-related quality of life^25^. The results of our study show that the adherence rate for OA treatment is approximately 68% at a mean follow-up period of 72 months which is higher compared to the 44% for CPAP in patients with comparable OSA severity^26^. Hence, OA treatment should not be ruled out for an OSA patient with headaches solely based on the modality’s efficacy in correcting certain PSG related sleep indices. The reason why improvement in headache with OA treatment was prevalent in the aspect of frequency needs to be further investigated. One can hypothesize that it may be due to our small sample size rather than being a result of the limited efficacy of OA in controlling headache severity. Although the improvement was majorly in overall headache frequency, severest headache intensity also significantly decreased in the headache responder group. Decrease in headache frequency itself is known to lead to a significant improvement in patient quality of life^27^ and based on such observations, headache frequency is also considered an important criterion in evaluating treatment success for headaches^28^. Overall, the patients with headache still had related symptoms after OA treatment but the severity and level of disability due to headaches showed a decrease. Such a trend is in line with previous results showing a reduction in temporomandibular disorders pain but not total eradication of symptoms following a combination of exercise and OA therapy^29^. Another factor to consider with OA treatment long-term is occlusal changes and its possible effect on residual headaches. Malocclusion and resulting oral parafunction could aggravate headache symptoms, however the specific type of malocclusion, protrusion of the lower incisors and retrusion of the upper incisors, that occurs with OA usage is variant from the condition generally associated with an increase in headaches^30^.

Patients with a unilateral headache had a higher possibility of not showing improvement in headaches following OA treatment for OSA. Unilateral headache is a distinct characteristic of neurovascular headaches that are generally known to have a stronger genetic component compared to tension-type headaches^31^. The controlling of contributing factors such as intermittent hypoxia and arousals through OA treatment may not have been sufficient in overcoming the inherent drive of headache in a migraine patient with OSA. Primary headaches have distinct characteristics regarding the underlying mechanism and aggravating factors so it is expected that treatment response and residual headache with OA treatment will depend on the type of headache the patient suffers from^32^.

Other pre-treatment characteristics including higher AHI, older age, higher BMI, and noncompliance to treatment were associated with being a headache non-responder after OA treatment. Such factors are also well known to predict OA treatment failure. This suggests the possibility that improvement in headache could truly be mediated through the alleviation of OSA symptoms which result from successful OA treatment^33^. Hence patient selection should be based on already reported predictors of success for OA treatment in OSA even in the aspect of headache management.

The presence of morning headaches showed a decrease in both groups with OA treatment. Morning headache is a cardinal sign of headaches due to OSA and is significantly more prevalent in patients with moderate to severe OSA. Also, morning headache is closely related to a lower oxygen saturation level during sleep. On the other hand correlation analysis results of this study showed that a longer time spent in < 90% oxygen was related to a lower headache intensity pre-treatment, implicating that intermittent hypoxemia could have different effects on various aspects of the headache. A previous study reported resolution of morning headache in 90% of patients treated with CPAP^34^. The resolution rate in our study was 50% in the headache responder group which is relatively low compared to results with CPAP. However, based on such observations, morning headaches may be considered as a diagnostic criterion to identify headaches due to OSA and eventually evaluate treatment success following OA treatment for headaches in OSA patients. The underlying mechanism of morning headaches cannot be determined based on the results of this study but the data shows that headache non-responders have more morning headaches before and also after OSA treatment while headache non-responders also have a higher AHI and lower lowest oxygen saturation value regardless of evaluation time. Among suggested mechanisms, hypoxemia has been reported to cause morning headache in OSA patients and our findings support its pathological role in the generation of morning headache^18^. Disruption in sleep architecture is another hypothesized contributor of morning headache, however there are studies that contend its irrelevance^35^. Our results support these studies showing that neither sleep efficiency nor percentage of each sleep stage showed significant differences between headache responders and non-responders. On the other hand, several PSG parameters related to sleep stage and efficiency showed a significant correlation with headache intensity and episode duration. Future studies comparing the effect of treatment between patients with only morning headaches and those with chronic headaches throughout the day are necessary to further speculate the mechanism of morning headaches and treatment effects.

PLM was more evident in headache non-responders and interestingly the index increased in both groups after OA treatment. Studies showed that children with migraine present a high PLM index which is related to more frequent and severe headaches that are refractory to headache treatment^36,37^. Restless legs syndrome was proposed as a cause of sleep disruption in primary headache patients since PLM is a powerful disruptor of sleep macrostructure leading to the deterioration in the restorative effect of sleep^38^. PLM may further aggravate headache symptoms in OSA patients through further fragmentation of sleep although it is difficult to explain the increase in PLM index following OA treatment. PLM index has a potential value considering its close relation to refractory headaches, so it should be evaluated through PSG especially when the OSA patient reports failure in headache management with conventional approaches.

There are a few limitations of this study due to its retrospective study design. A certain subset of OSA patients with headache were specifically included in the study group. Also, by only including patients that had agreed to and were eligible for OA treatment, the study group may have resultantly become to consist of patients with less severe types of OSA and headaches, thus not reflecting the general population that may have more severe OSA and other headache types. This could have again affected the results by excluding a patient population with certain demographic, psychological and clinical characteristics^39^. The change in headache characteristics was based on self-reported questionnaires without a direct clinical evaluation. However, the questions and scale applied in this method are well verified and reliable^40^. Another factor is the relatively small sample size and ethnicity bias which may have underestimated the response to OA treatment. The small sample size was due to the application of a strict diagnostic process based on level 1 PSG and exclusion criteria along with a high nonresponse rate to headache re-evaluation. Randomized trials based on larger study groups of OA treatment are necessary to fully elucidate the effect of OA on headache patients with OSA. Unrandomized studies evaluating treatment results based on CPAP compare patients who are tolerant or intolerant to treatment, which may result in bias since patients that are compliant to one therapy are more likely to be compliant with other therapies. To overcome such shortcomings, the patients in this study were mainly differentiated according to treatment response rather than treatment adherence.

Studies investigating headache patients with OSA should be based on level 1 PSG evaluations such as was with this study. Screening with portable sleep monitors could underestimate the severity and presence of sleep apnea and would result in overlooking patients with a relatively low respiratory index but still may benefit with their headaches after OA treatment. Also, without an electroencephalogram evaluating arousals and sleep architecture that may have a significant effect on headache it is difficult to verify sleep characteristics that are predictive of a headache responder following OSA treatment with OA.

This study is the first to report the effects of OA treatment in OSA patients with headaches. The results tentatively suggest that treatment with an OA is beneficial for many OSA patients with headaches. Although the exact causality between OSA and headache remains uncertain, headache patients should have their sleep, including daytime sleepiness and habitual snoring, evaluated in the diagnostic process and PSG should be considered if sleep apnea is suspected. Based on the data from this relatively small scale study, OA treatment could be applied in headache patients with mild to moderate OSA when the patient is an appropriate candidate or has failed with other OSA treatments including CPAP to further contribute to the improvement of headache symptoms.

## Methods

### Participants

Initially, 150 consecutive adult (≥ 18 years) mild‐to‐severe OSA (apnea–hypopnea index [AHI] ≥ 5 events/h) patients who were referred to the Sleep Apnea Dental Clinic at the University of British Columbia (UBC) or to an affiliated private practice from September, 2004 to June, 2007 for OA treatment were invited to participate via mail. Among those 72/150 (48%) reported to have headaches, among which 47/72 (65%) accepted to take part. Finally, 13/72 (18%) participants who completed the headache reevaluation among which 8/72 (11%) participants had follow-up PSGs were included in the final analysis. Those with an initial diagnosis of moderate‐to‐severe OSA (AHI ≥ 15 events/h) or mild OSA (AHI ≥ 5 events/h) with associated symptoms including excessive daytime sleepiness were included only after the patient failed or refused to try CPAP treatment. All participants underwent clinical and PSG evaluation for OSA diagnosis and to verify their eligibility for OA treatment.

Patients were excluded from the study if he/she did not report any headache symptoms and when refused or was not appropriate for OA treatment due to advanced periodontitis, dental caries requiring treatment, active temporomandibular joint disorders pain either from the masticatory muscles and/or temporomandibular joint (TMJ) and/or severe mouth opening limitation with a maximum mouth opening range < 30 mms, and less than six remaining posterior teeth. Also those with uncontrolled psychological, cardiovascular or respiratory disease, pregnancy, acute or chronic systemic inflammatory disease, a body mass index (BMI) > 35 kg/m^2^, previous OA treatment for OSA and non-adherence with the study protocol were excluded.

Final patient grouping was based on the reduction of headache frequency (> 30%) following OA treatment.

The study conformed to the principles outlined by the Declaration of Helsinki and was approved by the UBC Clinical Research Ethics Board (#H09-01920). Informed consent was obtained from all participants prior to recruitment.

### Oral appliance treatment

All patients were fitted with a mandibular advancement device (Klearway, Space Maintainers Laboratories Canada Ltd., Calgary, Canada). The appliance was custom made with a titratable design to cover the occlusal surfaces of all upper and lower teeth. Semi-rigid thermoplastic material was used for its construction. The amount of initial advancement was set at two-thirds of the possible amount of maximum protrusion for each patient, and then further advancements were prescribed by 0.25 mm increments until self-reported resolution of snoring and related symptoms such as daytime sleepiness. The advancement was also stopped when the patient complained of any type of discomfort due to the appliance. The vertical opening was kept to a minimum of approximately 3–5 mm to avoid downward rotation of the mandible during use. Optimal titration was then verified by a follow-up sleep study. Patients that were comfortable with their OA after 1 month were scheduled for recall checks at 6 months, 1 year, and 2 years after wearing the appliance.

Treatment success in the aspect of AHI improvement was defined as (a) resolution of OSA (AHI < 5 events/h); (b) success (AHI ≤ 10 events/h); (c) suboptimal (10 < AHI ≤ 20 events/h); or (d) failure (AHI > 20 events/h). Patients were also differentiated as OSA responder (a decrease in AHI of ≥ 50% from baseline) and non-responder (decrease < 50% or increase in AHI from baseline).

Treatment adherence was measured by self-reported questionnaires based on how many nights per week (every night, 4–6 nights, 1–3 nights, less than once) and how much of a single night (all night, more than half, half, less than half of the night) the patient used the appliance. Criteria for regular use of an appliance was defined as using the appliance for > 50% the night on ≥ 4 nights a week^41,42^.

### Headache evaluation

All patients completed the Migraine Disability Assessment Questionnaire (MIDAS) at the initial visit^40^. Their disability level was expressed as the MIDAS total score. The patients were differentiated into a low (MIDAS score 0–10) or high (MIDAS score ≥ 11) disability group based on this score. Also participants answered a headache characteristics questionnaire including items on frequency (number of episodes during the past 3 months), intensity (average and severest during the past 3 months and current) on a 0–10 visual analog scale (VAS, 0 is no pain and 10 is the worst imaginable pain possible), timing (morning, afternoon, evening, and middle of the night), duration, aggravating and alleviating factors, localization (right, left, both sides), family history of headaches, and concomitant conditions including nausea and vomiting, sensitivity to light/sound/smell, and autonomic symptoms (conjunctival tearing, rhinorrhea, facial sweating, ptosis). The headache type was determined based on the patient’s symptom history in accordance with the International Headache Society International Classification of Headache Disorders (ICHD-2)^43^.

Follow-up evaluation was done with a questionnaire following completion of OA titration and a usage duration of 5.7 ± 1.1 (mean ± SD) years with identical items evaluated at the initial visit. However, questions asking the persistence of headache symptoms (do you still suffer from headaches?) and adherence to OA therapy were added to the initial questionnaire. Improvement in headaches was assessed by comparing pre- and post-treatment headache questionnaire results. Patients were grouped into headache responder and non-responder according to the presence of a > 30% reduction in headache frequency following OA treatment and the final analysis was based on such grouping^28^.

### Polysomnographic evaluation of OSA

Attended standardized PSG was performed in the hospital using Embla Sandman Software (Natus Medical, Inc., Pleasanton, CA) and scored according to the American Academy of Sleep Medicine (AASM) criteria^44^. Electroencephalogram, electro-oculography, electrocardiogram, oxygen saturation, airflow using nasal pressure, leg and chin electromyography, chest and abdominal movement, and snoring were measured. To analyze sleep architecture, parameters such as total sleep time, sleep efficiency, sleep onset latency, wakefulness after sleep onset (WASO), rapid eye movement (REM) latency, as well as the percentage of each sleep stage were recorded. An apnea was defined as a > 90% amplitude decrease of the nasal pressure signal from baseline for ≥ 10 s. Hypopneas were a > 50%, but < 90% decrease from baseline, or a clear amplitude reduction of the nasal pressure signal that did not reach the above criterion but was associated with either an oxygen desaturation ≥ 3% or an arousal, and lasted for ≥ 10 s. AHI was defined as the number of apnea and hypopnea events/h, AI was the number of apneic events/h, and HI was the number of hypopnea events/h. The mean and longest duration of apnea and hypopnea events, respiratory related and spontaneous arousals, and positional dependency and REM relatedness of the respiratory events were evaluated. Lowest oxygen saturation (LSAT), mean oxygen saturation, and the percentage of time spent below 90% oxygen saturation were recorded. Periodic limb movements (PLM) were also recorded and analyzed as the periodic limb movement index.

OSA severity was differentiated into mild (AHI 5–14 events/h), moderate (AHI 15–29 events/h), or severe (AHI ≥ 30 events/h). Positional OSA patients were defined as those with a supine AHI to non-supine AHI ratio > 2^45^. REM-related OSA patients were defined as those with a non-REM (NREM) AHI < 15 events/h, and a REM AHI to NREM AHI ratio > 2^46^. Hypopnea/apnea ratio (HAR) was also calculated from the results^47^. Pre-treatment PSG was performed at the latest 1 month before treatment initiation. Eight participants underwent post-treatment follow-up level 1 PSG studies within 1.9 ± 2.5 years (mean ± SD) of OA initiation.

On the night of the initial PSG, patients completed questionnaires with items related to medical and psychiatric history, sleep habits, health habits including smoking, caffeine, and alcohol intake, sleep symptoms, and medications. Weight and height were measured and BMI was calculated as weight in kilograms divided by the square of height in meters. Daytime sleepiness was assessed with the Epworth Sleepiness Scale (ESS), a validated, self‐administered questionnaire. ESS ≥ 10 was considered as having excessive daytime sleepiness^48^.

### Statistical analysis

Kolmogorov–Smirnov and Shapiro–Wilk tests were used to test the normality of data and each following test was selected accordingly. Differences in demographic and clinical parameters concerning headache and polysomnographic characteristics based on the response in headache status were analyzed by t-test, Mann–Whitney test, and Chi-square test. Differences in clinical parameters concerning headache and polysomnographic characteristics before and after OA treatment in each headache response group were analyzed by paired t-test, Wilcoxon Rank-sum test, and McNemar’s test. Differences in clinical parameters concerning headache based on OA adherence were analyzed by t-test, Mann–Whitney test, and Chi-square test and before and after OA treatment in each adherence group were analyzed by paired t-test, Wilcoxon Rank-sum test, and McNemar’s test. Correlations of pre- and post-OA treatment headache parameters and pre-treatment polysomnographic parameters were analyzed by Spearman’s correlation coefficient. All statistical analysis was performed using SPSS 22.0 (IBM, Chicago, IL). Results were considered statistically significant when p < 0.05.
